# Examining Effects of Anticipated Stigma, Centrality, Salience, Internalization, and Outness on Psychological Distress for People with Concealable Stigmatized Identities

**DOI:** 10.1371/journal.pone.0096977

**Published:** 2014-05-09

**Authors:** Diane M. Quinn, Michelle K. Williams, Francisco Quintana, Jennifer L. Gaskins, Nicole M. Overstreet, Alefiyah Pishori, Valerie A. Earnshaw, Giselle Perez, Stephenie R. Chaudoir

**Affiliations:** 1 Department of Psychology, University of Connecticut, Storrs, Connecticut, United States of America; 2 Center for Interdisciplinary Research on AIDS, Yale University, Hew Haven, Connecticut, United States of America; 3 Department of Psychology, College of the Holy Cross, Worcester, Massachusetts, United States of America; Massachusetts General Hospital, United States of America

## Abstract

Understanding how stigmatized identities contribute to increased rates of depression and anxiety is critical to stigma reduction and mental health treatment. There has been little research testing multiple aspects of stigmatized identities simultaneously. In the current study, we collected data from a diverse, urban, adult community sample of people with a concealed stigmatized identity (CSI). We targeted 5 specific CSIs – mental illness, substance abuse, experience of domestic violence, experience of sexual assault, and experience of childhood abuse – that have been shown to put people at risk for increased psychological distress. We collected measures of the anticipation of being devalued by others if the identity became known (anticipated stigma), the level of defining oneself by the stigmatized identity (centrality), the frequency of thinking about the identity (salience), the extent of agreement with negative stereotypes about the identity (internalized stigma), and extent to which other people currently know about the identity (outness). Results showed that greater anticipated stigma, greater identity salience, and lower levels of outness each uniquely and significantly predicted variance in increased psychological distress (a composite of depression and anxiety). In examining communalities and differences across the five identities, we found that mean levels of the stigma variables differed across the identities, with people with substance abuse and mental illness reporting greater anticipated and internalized stigma. However, the prediction pattern of the variables for psychological distress was similar across the substance abuse, mental illness, domestic violence, and childhood abuse identities (but not sexual assault). Understanding which components of stigmatized identities predict distress can lead to more effective treatment for people experiencing psychological distress.

## Introduction

People with concealable stigmatized identities – socially devalued identities that can be hidden from others – show great variability in their experience of psychological distress. Some people are quite resilient while others suffer greatly. The goal of the current work is to examine the extent to which beliefs and experiences related to the stigmatized identity can predict variance in psychological distress. We attempt to replicate and then extend previous research on concealable stigmatized identities. Specifically, we will address the following: (a) Utilizing a low-SES, diverse, community sample of adults, we will test replication of research conducted with college students showing that anticipated stigma, centrality, and salience predict psychological distress [1]; (b) We include two additional stigma variables in our prediction model: stigma internalization and level of outness of the identity. Testing these 5 stigma constructs simultaneously will help give clarity to a research field that often uses the constructs interchangeably; (c) Unlike previous research where many different concealed stigmatized identities were lumped together or only one identity was examined, our sample includes 5 distinct concealed stigmatized identities—mental illness, substance abuse, sexual assault, domestic violence, and childhood abuse–that we can compare. We will examine both between group differences in mean level of stigma experience as well differences in ability of the stigma variables to predict distress across the groups.

Research on a diverse range of concealable stigmatized identities such as mental illness, sexual assault, childhood sexual abuse, HIV-status, and minority sexual orientation has found increased levels of psychological distress among these groups compared to their non-stigmatized peers [2]–[5]. Although measuring between group differences in level of distress is important, it is crucial to not lose sight of the immense within group variability that exists. Drawing on research and theory on specific concealed identities such as minority sexual orientation (minority stress model [2]), mental illness [6], and HIV+ status [7], we have been developing and testing a more general model of how concealable stigmatized identities affect psychological distress [1], health outcomes, and behaviors [8]–[10].

In the current work, we focus on predicting level of psychological distress, as measured by a composite of depression and anxiety. Psychological distress is important for several reasons. Anxiety and depression are the two most common psychological disorders, affecting nearly a quarter of the adult U.S. population, and yet among those who are diagnosed only about a third receive adequate treatment [11]–[12]. The consequences of untreated anxiety and depression can have profound implications at individual, family [13]–[17], community [18], and societal levels. Indeed, the National Institute of Mental Health [19] estimated that the costs of mental health care service totaled $57.5 billion in 2006, making it the third most costly medical condition (tied with cancer).

### Concealable Stigmatized Identity (CSI) and Predicting Distress

In the current work we examine the ability of 5 stigma identity constructs to predict psychological distress for people with concealable stigmatized identities (CSIs). Although many variables (i.e., environmental, genetic, interpersonal, dispositional, structural) account for variance in distress, in the current work we focus on cognitions and experiences related to the stigmatized identity by the person with the CSI. Based on our theoretical model, we divide our variables into two types (see [20] for full model description). First, constructs with emotional valence are those that are related to specific experiences or beliefs about the identity and contain affective and evaluative components, including anticipation of future stigma, internalization of negative stereotypes about the identity, and level of “outness” or disclosure to others. Second, constructs that capture the magnitude of the identity within the self include identity centrality and identity salience. These two constructs measure the size and meaningfulness of the identity to the self but not the emotional valence. This set of predictors allows us to examine a full range of identity experiences, beliefs, and meaningfulness. Below, we detail each construct and how we hypothesize the relationship to distress in the current research.

### Measuring Identity Valence: Anticipated Stigma, Internalization, and Outness

People with CSIs are likely to have a variety of different experiences and beliefs related to their identity. *Anticipated stigma* is the extent to which a person believes it is likely that others will devalue or distance themselves from the person with the CSI if the identity becomes known. Previous work has shown that anticipated stigma is a strong predictor of psychological distress among college samples with a variety of different CSIs [1]. Anticipated stigma within the health care system predicts people avoiding or underutilizing needed health care services [8]. Whether people anticipate stigma or not, they may have internalized the negative stereotypes about their CSI simply by living in a society that denigrates them. *Stigma internalization* occurs when people believe the negative stereotypes about their identity to be true of the self and/or wants to reject and distance the self from the identity. Internalized stigma has been related directly to psychological distress for people living with HIV-AIDS [21]–[22], for people with mental illness [23]–[24], and for LGB individuals [25]. Thus it has been a strong predictor of distress throughout the literature, and we, too, predict that greater internalization will be related to greater psychological distress.

Whereas most research on stigma focuses on negative experiences, there are identity related constructs that may be more positive and predictive of less distress. One such construct is *outness* or the extent to which other people in the environment know about the identity. Research on outness with LGB and HIV+ samples generally shows that being more out is related to less psychological distress [26]–[27], although there can be important moderators, such as the perceived supportiveness of the environment [28] and individual level of rejection sensitivity [29]. A recent review of strategies to reduce self-stigma of mental illness promotes being more out about one's mental illness history as a way to increase self-esteem and decrease self-stigma [30]. A pilot study supports the positive effects of outness for people with mental illness [31]. Thus, we predict that greater levels of outness will be related to lower levels of psychological distress.

### Measuring Identity Magnitude: Centrality and Salience

*Centrality* is the extent that an identity is considered central and important to one's self-definition. Given the variability in the types of CSIs – rape, substance abuse, childhood abuse – the centrality of the identities is also likely to vary widely. Some people may think of the CSI as a single life experience that is just a small part of their identity whereas others may find the same identity to be largely self-defining. Centrality has been a key construct in work on visible stigmatized identities, particularly racial identity. Research on centrality and racial identity among ethnic minorities has shown inconsistent results with some evidence that greater centrality is associated with less psychological distress [32], more psychological distress [33], or is unrelated to distress [34]. This pattern has been reconciled to some extent by research showing that identification with a racial identity is a personal resource that people can use to deflect perceived devaluation of their group [35]. When centrality of racial identity is perceived as a positive self-resource, it is correlated with feelings of solidarity, similarity, and satisfaction with the identity [36]. We do not think this positivity can be assumed for the concealable stigmatized identities we are examining because people with CSIs often lack access to similar others and positive group-level identities [37]. Indeed, previous work on concealed identities found greater centrality was related to more distress [1]. Thus, in the current work we expect greater centrality of the CSI to predict greater reported distress.

As an additional measure of identity magnitude, we include *salience*, or the frequency with which a person is thinking about the identity. Salience is not meant to capture whether the thoughts about the identity are positive or negative, but rather the frequency of thoughts. In essence, a person could conceptualize a CSI as not being defining of the self (low centrality), but may nonetheless spend a lot of time thinking about the identity (high salience). In previous work, salience predicted psychological distress and it did so even when controlling for levels of anticipated stigma and centrality [1]. Salience may capture the cognitive burden of thinking about an identity that is kept secret [38]. In the current work, we hypothesize that increased salience will predict increased distress.

### Examining 5 Specific Concealable Stigmatized Identities

Work on CSIs has largely proceeded by examining separate identities in isolation. There is a body of research, for example, on mental illness stigma, both in the sociology and psychology literatures [23], [39]. Separate from that, often using completely different terminology and measures, is stigma work on minority sexual identity [2]–[3]; on HIV+/AIDS stigma [40]–[42]; on epilepsy stigma [43], and domestic violence [10], [44], amongst others. In our work we have tried to formulate a general model that incorporates key constructs from these various literatures as well as the large body of work with visible identities [1], [20], [45]. Despite working to build a general model, we recognize that the model may operate differently for different identities but we have been heretofore unable to examine this question. In the current research we collected data from people with five specific CSIs, with the goal of being able to conduct analyses both with the full group and with each individual group, allowing further insight into how stigma variables may (or may not) predict distress within specific identities.

In the current research project we focus on five CSIs: mental illness, experience of childhood abuse (physical, emotional, and sexual), experience of domestic violence, experience of sexual assault, and substance abuse. We chose these five identities for four reasons. First, they are very common, affecting a large part of the population, thus the research is maximally useful. For example, 14–32% of adults report a history of childhood sexual abuse [46], 8.8% of adults meet criteria for substance dependence or abuse (alcohol and illicit drug) in the past year [47], rates of domestic violence range from 10–30% of married couples [48] and are reported at nearly equal rates for men and women [49], and 26% of adults have experienced some type of mental illness [11]. Second, they are stigmatized identities, both in the sense that having such an identity is seen as a mark of failure or shame as well as being experienced as something that devalues the self in the eyes of others and should be hidden (e.g., for mental illness [39], [50], for rape [51]–[54], for domestic violence [55]–[56], for substance abuse [57]–[61], for childhood sexual abuse [62]–[64]). Third, they decrease the likelihood of having a disproportionate representation of one gender. Although we expect to see a higher percentage of men reporting substance abuse and women reporting rape; mental illness, child abuse, and domestic violence occur with a high degree of frequency for both genders. Fourth, these 5 identities have all been related to increased psychological distress, yet variability exists in how people are affected. The risk of depression, anxiety, trauma related symptoms, poor health outcomes, reduced quality of life, disability burden, co-morbidity and dual diagnosis, and interpersonal difficulties is substantially increased for these stigmatized identities [65]–[67]. In addition, lack of effective treatment and impaired functioning over time are associated with increased distress, poorer outcomes, and risk for revictimization [68]. Yet, despite the increased risk of psychological distress and negative consequences, there is significant variability in how these outcomes are experienced between, across, and within individuals [69]–[70]. In short, although we are testing our theoretical predictions, we are also attuned to the clinical impact of our findings.

Regardless of whether people with CSIs believe negative stereotypes about their groups, they are aware of societal stigma and devaluation [71]. The five identities in the current work do vary in the extent to which they are culturally devalued and this may impact the mean levels of anticipated stigma, internalization, centrality, salience, and outness that people report. Research from an attributional perspective finds that people whose stigmatized identities are considered controllable – either in their onset or in their continuation – elicit more negative affect and blame from others compared to identities considered less controllable [72]. Research has found that substance abuse and mental illness are considered personally controllable [72]–[74], although blaming the victims for sexual assault and domestic violence also occurs [10], [75]. Mental illness and substance abuse are also more likely to elicit dispositional attributions because they do not include the “perpetrator/victim” context associated with child abuse, sexual assault, and domestic violence. In addition to attributions of blame, evolutionary psychology suggests that certain people are stigmatized because they would make poor exchange partners or members of cooperative groups [76]. People considered dangerous or unpredictable are such poor exchange partners. Here again, a consistent stereotype of people with substance abuse and mental illness is their dangerousness and unpredictability [74], [77]. Thus, from both attributional and evolutionary perspectives, substance abuse and mental illness are more culturally stigmatized than the other three identities. Given these differences, we expect that participants with mental illness and substance abuse identities will report increased anticipated and internalized stigma. There is, however, no a priori reason to predict differences in centrality, salience, or outness across the 5 groups.

## Overview

In the current research, we collected data from an adult community sample. We sought a sample that was quite different from the university samples used in previous work. Participants report on one of five CSIs: Mental illness, substance abuse, domestic violence, sexual assault, or experience of childhood abuse. Using the full sample, we will first examine whether we replicate that anticipated stigma, centrality, and salience each uniquely predict psychological distress. We will then add internalized stigma and outness to examine if they account for additional variance in distress. As noted above, we predict that anticipated stigma, centrality, salience, and internalized stigma will uniquely predict increased distress whereas outness will predict decreased distress.

Next, we will examine interactive effects. We hypothesize that the magnitude of the identity should moderate the effects of the valenced content. Thus, for people whose identity is relatively small in magnitude (low centrality or salience), the effects of negative valenced content (anticipated stigma or internalization) on distress should be attenuated; whereas for people whose identity is larger in magnitude (greater centrality or salience), the effects of negatively valenced content should be greater. In addition, because anticipated stigma is a worry about being rejected if others find out about the identity and previous research has shown that the benefits of outness for gay men are moderated by level of rejection-sensitivity [29], we will examine whether outness and anticipated stigma interact such that for people high in anticipated stigma, the relationship between outness and reduced distress is attenuated.

Once we have examined the direct and interactive effects of the stigma variables on distress for the full sample of CSIs, we will examine the mean level differences in the stigma variables for each of the 5 types of CSI. As noted above, we expect that people reporting on mental illness and substance abuse will report greater levels of anticipated and internalized stigma because these identities are more culturally devalued. There is no *a priori* reason, however, to expect that there would be differences in centrality, salience, or outness across the five identities. Finally, we will examine whether the direct effects model (with all 5 stigma variables predicting distress) differs across the 5 identities. Because we developed the model to be general, we do not expect it to be moderated by identity type.

## Method

Data were collected over three years (2009–2011) from three locations in and around Hartford, Connecticut. Locations were chosen to maximize the probability of reaching participants with one of the target concealed identities. Locations included (1) a state run agency offering mental and behavioral health care counseling; (2) a private community-based agency offering a range of social services (e.g., housing and employment assistance, counseling) to predominately African American and Latino communities; and (3) a community college that serves a diverse student population with higher representation of racial/ethnic minorities, veterans, and transitioning students than other local colleges.

### Ethics statement

All study procedures and measures, including consent and debriefing forms, were approved by both the institutional review board of the University of Connecticut and the institutional review board of the Department of Mental Health and Addiction Services (DMHAS) of the State of Connecticut.

Because our study focused on identities that are both stigmatizing and concealable, considerable time was devoted to training and supervising research assistants to ensure that data were collected in a sensitive and consistent manner and that participant identities were not inadvertently exposed. To read a full description of the experimenter training see Text S1.

### Procedure

At each location one or more trained research assistants approached people in public areas using a prepared script and wearing badges identifying their affiliation with the university conducting the research. Potential participants were first asked if they were at least18 years old. If they were not 18, they were not able to participate in the study. However, when the data were reviewed it was found that some participants wrote in response to the open-ended age question that their age was 17. On advice from the Institutional Review Board, data from these participants were deleted from the dataset.

If participants were interested in the study, the assistant led them to a room set aside for the study to complete the survey on a mini-laptop using MediaLab software [78]. Use of the laptop and mouse was explained and the consent form was read aloud by the experimenter while the participant viewed it onscreen. Participants were given a choice of completing the survey in English or Spanish and at least one bilingual research assistant was available at all times. Because there was not an option to have the full survey read aloud, participants needed to be literate. To check for the necessary literacy level, there were several example questions in the beginning of the survey devised so that participants had to be able to read the directions in order to answer the question correctly. These questions also served to explain the way the scales worked. If a participant could not answer the questions correctly, they were asked to stop the survey and thanked for their interest. This occurred for 2 participants. Upon completion of the survey, participants were given a sheet explaining the purpose of the study that also included resources for mental health, substance abuse, and shelter services if needed or desired. They were paid $15–20 for survey completion.

#### Participant Demographics

We surveyed 735 people total. The first prescreening question asked participants if they had any of the 5 target CSIs. If participants clicked on any of the CSIs to signify they had it, they were taken to a second question that asked them to choose the CSI that was most important to them. The computer program entered this specific identity into all of the questions pertaining to the concealed identity, thus the survey was personalized to each participant's identity. Because participants did not have the option of choosing multiple stigmatized identities, we do not have a count of how many participants had more than 1 of the 5 target identities. In total, 394 participants signified they had one of the target identities. The sample of CSIs included N = 105 people reporting on mental illness, N = 103 on substance abuse, N = 74 on experience of childhood abuse (physical, sexual, or emotional), N = 65 on domestic violence (experiences of physical abuse from a partner), and N = 47 on experience of sexual assault. Demographic information for the CSI sample is included in Table 1.

**Table 1:** Demographics.

Concealable Stigmatized Identity (CSI)	
Mental Illness	26.6%
Substance Abuse	26.1%
Experience of Childhood Abuse	18.8%
Experience of Domestic Violence	16.5%
Experience of Sexual Assault	11.9%
Age (in years)	32.18 ±11.85
Sex	
Male	58.4%
Female	41.6%
Education	
Did not complete high school	31.6%
Completed High School	24.9%
Some College or Technical Program (did not complete)	29.9%
Completed 2-year or Technical Program	7.6%
Completed undergraduate degree or above	6.0%
Income	
Less than $10,000 per year	60.9%
Between $10,000 and $20,000	15.8%
Between $20,000 and $50, 000	11.9%
Over $50,000	11.1%
Currently Employed	31.2%
Ethnicity	
Hispanic	40.1%
Non-Hispanic	59.9%
Race (Can choose multiple categories)	
African American	30.5%
White	29.7%
Asian or Pacific Islander	2.5%
Native American	2.8%
Other	34.3%
Language of Survey	
English	86%
Spanish	14%

Notes: In accordance with federal guidelines, participants were asked their race and ethnicity separately. Ethnicity was asked as a dichotomous choice between Hispanic and not Hispanic. Participants could choose multiple racial identity categories.

### Measures: Concealed Stigmatized Identity

**Anticipated Stigma** captures people's concerns about mistreatment and devaluation from others if their concealed identity becomes known. Starting with the stem “If others knew about your experiences of {*specific CSI inserted here*}, how likely do you think the following would be to occur?” 15 items were presented, 9 of which were taken from the “day-to-day” discrimination scale of Kessler, Mickelson, and Williams [79] (e.g. “People acting as if you are not smart,” “You are threatened or harassed”), as well as 6 additional items focusing on more relational concerns (e.g. “Friends avoiding or ignoring you,” “People not wanting to date you”). Each question is on a scale of 1 (Very Unlikely) to 7 (Very Likely). This 15-item scale was used previously in Quinn and Chaudoir [1]. The scale has high internal reliability, with an alpha of.95.

**Centrality** or the importance of the identity to the self was initially measured with 8 items – 4 items from the Identity subscale of the Collective Self-Esteem scale [80] plus 4 additional items created for this study. Two items from the Collective Self-Esteem scale are reverse worded (e.g., “My {CSI} is not important to my sense of what kind of person I am.”), and these items were confusing to participants (item to total scale correlations after being reversed were −.06 and −.21) and reduced reliability for the 8-item scale to.62. Thus, a 6-item scale with all questions asked in the same direction, with an alpha of.81 was retained. The items are as follows, all on a 7 point scale of strongly disagree (1) to strongly agree (7): “My {CSI inserted} is an important reflection of who I am,” “In general, my {CSI} is an important part of the way I see myself,” “My {CSI} defines who I am,” " It is impossible to understand me without knowing about my {CSI},” “I would be a different person without my {CSI},” and “My {CSI} is a central part of my self-definition.”

**Salience** is the frequency with which people are thinking about their CSI. We used 3 items to capture salience. The first item was used previously [1]: “How often do you think about your {CSI}?” with a scale ranging from almost never (1) to many times each day (7). We included two additional items “I spend a lot of time thinking about my {CSI}” and “My {CSI} often crosses my mind for no reason.” Both were measured on a 7 point scale from strongly disagree (1) to strongly agree (7). This 3-item scale has a Cronbach's alpha of.78.

**Internalization** of the negative beliefs about the self was measured with 4 items, based on Link's Devaluation-Discrimination scale for mental illness [71] with modification [81]. The scale had a Cronbach's alpha of.78. The items were “I feel that my {CSI} is a sign of personal failure,” “I would not want to date someone with {CSI},” “Most of the negative things people think about {CSI} are true,” and “I don't blame people for wanting to keep their distance from me when they find out about my {CSI}.” All items were asked on a Strongly Disagree (1) to Strongly Agree (7) scale.

**Outness** was measured using a 1-item scale modified from Cole, Kemeny, and Taylor [29]: “Relative to other people with {insert CSI} would you consider yourself: (1) Definitely in the Closet (Almost nobody knows about the identity), (2) In the Closet most of the time (Most of the time, the people around you are not aware of the identity), (3) Half in the closet, half out of the closet (People around me are not aware of my identity about half the time), (4) Out of the Closet most of the time (Most of the time, people around me know my identity), (5) Completely out of the closet (Just about everybody knows my concealed identity).”

### Measures of Psychological Distress

**Psychological Distress** was measured with two scales: The 20-item Center for Epidemiological Studies-Depression scale (CES-D; [82]) and the 20 item Spielberger Trait Anxiety Scale (STAI-T; [83]). Both scales are well validated with adult samples. Questions on the CES-D are asked based on the frequency of symptoms over the last week on a 0 (Rarely or None of the Time [Less than 1 Day]) to 3 (Most or all of the Time [5–7 Days]) scale. Although sums rather than averages are often reported in the literature for the CES-D, we had a programming glitch for the first 74 participants in the study where one of the scale items did not appear. Because these participants only answered 19 items instead of 20, reporting sums would result in errors. As a result, we use averages rather than a sum score. Using an alternative scoring method for the CES-D has been employed by other researchers [84]. For the STAI, participants are asked to report the frequency of symptoms based on how they “generally feel.” Each item is on a 1 (almost never) to 4 (all the time) scale. Each scale is high in internal validity. For this study, Cronbach's alpha is.90 for the CES-D and.89 for the STAI-T. The two scales are correlated at.79. In order to create the composite measure of psychological distress, all items are standardized and then aggregated. The 40-item scale has an alpha of.94.

**Neuroticism/Emotional Stability** was measured in order to control for a personality variable that is associated with reporting increased distress. We used the 1 item emotional stability from the Single-Item Measures of Personality (SIMP; [85]). The SIMP has shown good convergence with longer measures of personality. The bipolar measure ranges from 1 to 9 with higher numbers indicating greater neuroticism. Mean level of neuroticism is 5.63 (SD  =  2.32).

## Results

### Predicting Distress for the Full Sample of CSIs

Table 2 displays the zero-order bivariate correlations between all of the stigma variables and distress (as well as means and standard deviations for each variable). As would be predicted, anticipated stigma, centrality, salience, and internalization are each correlated positively with distress; whereas outness is negatively correlated with distress. The effect sizes of the relationship between each of the stigma measures and distress range from small (outness and centrality) to medium (anticipated stigma, salience, and internalization). Although the stigma variables are correlated with each other, the correlations range from.05 to.53, demonstrating construct validity but no problems with discriminant validity. Nonetheless, it is only by examining the variables simultaneously in regression that we will be able to ascertain whether each of these constructs accounts for unique variance in distress.

**Table 2:** Means (SD) and Zero-Order Bivariate Correlations for the Full Sample of Concealable Stigmatized Identities.

	Anticipated Stigma	Centrality	Salience	Internalization	Outness	Distress
Anticipated Stigma	1	.30**	.53**	.46**	.12*	.37**
Centrality		1	.44**	.23**	.07	.27**
Salience			1	.43**	.11	.44**
Internalization				1	.05	.31**
Outness					1	−.12*
Distress						1
Means (SD)	3.71(1.75)	3.87(1.56)	3.98(1.83)	3.17(1.57)	2.67(1.40)	.16(.54)

Notes: **p<.01; *p<.05.

Using hierarchical linear regression, we first examine a replication of the Quinn and Chaudoir [1] finding that showed anticipated stigma, centrality, and salience each accounted for unique variance in psychological distress. Including all participants with a CSI, we regress psychological distress first on the demographic factors: Income, education, and sex. Table 3 (Step 1 column) shows that together these demographic factors account for 5% of the variance in distress in this sample, with people with higher incomes and educational level showing lower levels of distress and women tending to report more distress. In the next step, we entered anticipated stigma, centrality, and salience. With anticipated stigma, centrality, and salience entered simultaneously in the model (see Step 2 in Table 3) we see a partial replication of Quinn and Chaudoir's [1] findings with college students: Anticipated stigma and salience account for unique, significant amounts of variance. Centrality however, is not significant. Thus, in this very different, diverse, low SES, community sample, the model largely replicates and accounts for 25% of the variance in psychological distress—roughly equal to the amount of variance accounted for in the college student sample [1].

**Table 3:** Predicting Psychological Distress for all Concealable Stigmatized Identities.

	Step 1	Step 2	Step 3	Step 4	Step 5
	β	β	β	β	β
Income	−.16*	−.12*	−.12+	−.13*	−.11+
Education	−.13*	−.11*	−.11+	−.09	−.10+
Sex	.07	.09+	.10+	.08+	.07
Anticipated Stigma		.18**	.15*	.17*	.16*
Centrality		.07	.07	.07	.08
Salience		.30**	.28**	.29**	.28**
Internalization			.09	.08	.09
Outness				−.17**	−.16**
Neuroticism					.15**
**Change in R^2^**	**.05** **	**.21** **	**.01**	**.03** **	**.02** **
**Total Adjusted R^2^ (and full model significance)**	**.05** **	**.25** **	**.26** **	**.29** **	**.30** **

Notes: Outcome variable is Psychological Distress.

**p≤.001,

*p≤.01, and ^+^ *p*≤.05.

We now turn to two additional stigma variables—internalization and outness—to test for further variance accounted for in distress. In Step 3 (Table 3), we added stigma internalization. The addition of internalization accounts for 1% of additional variance, but it is not statistically significant. Anticipated Stigma and salience remain significant when entered simultaneously with internalization. In Step 4, we added level of outness, or how open people are about their CSI with others in their environment. We expected outness to offer a level of protection, and, indeed, greater outness was related to less psychological distress, accounting for an additional 3% of the variance in distress. Notably, in this step anticipated stigma, salience, and outness are each unique significant predictors of distress. Taken together, the model accounts for 29% of the variance in psychological distress, a large effect size.

Finally, we address a critique of the stigma literature that perhaps what we are detecting is that the same people who are anticipating or perceiving stigma in the world are also more likely to report distress because of an underlying personality trait—not because of stigma, *per se*. In this case the relationship between stigma and distress would be spurious. In order to test this possibility, we added a measure of neuroticism in Step 5 (Table 3). People who report more dispositional emotionality (neuroticism) do report more distress. However, as can be seen by comparing the betas in steps 4 and 5, controlling for neuroticism had little effect on the relationships between the stigma variables and distress.

#### Interactions

Thus far we have been examining unique direct effects of the stigma variables. We also predicted that the magnitude and emotionally valenced content would interact. People who feel the identity is more central or salient *and* report more negatively valenced stigma (i.e., increased anticipated stigma or internalization) may experience more distress than those whose identity is lower in magnitude (less central or salient). Thus, magnitude may be a moderator of the relationship between valenced content and distress. In order to test these possible interactive effects, we first centered each of the stigma variables, and then we created product terms for each of the 2-way interactions. We ran a new regression analysis with distress regressed on the demographic variables in Step 1, the 5 stigma variables in step 2, and all 2 way interactions in step 3. Results showed that only one 2-way interaction was significant: Centrality by Internalization, β = .14, p = .04.

**Figure 1:**
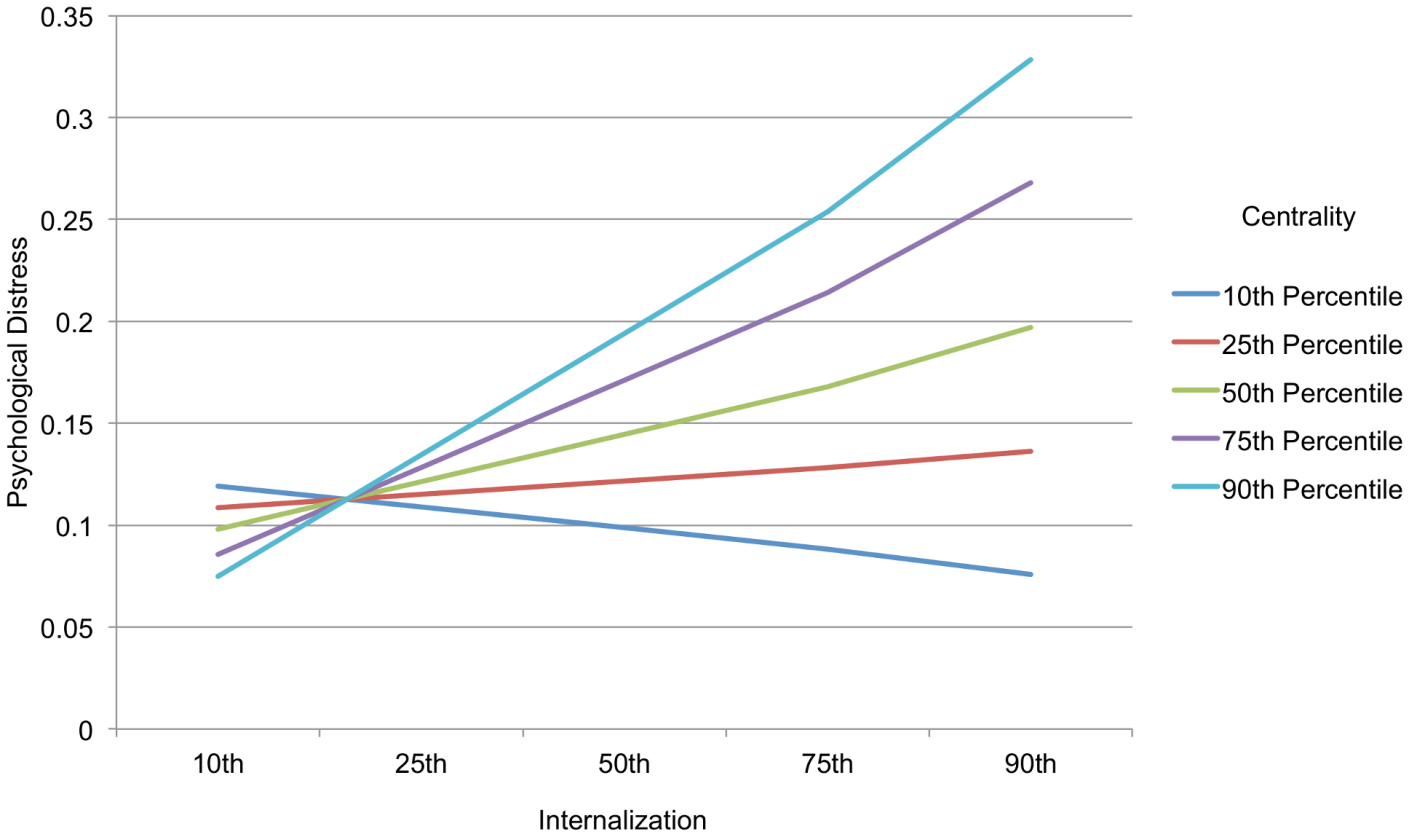
Interaction between Centrality and Internalization on Psychological Distress. At most levels of identity centrality (below the 75^th^ percentile), the relationship between internalization and psychological distress is not significant. At very high levels of centrality, however, greater internalization is related to greater psychological distress. Note: Plot based on predicted values of distress given values of the predictor variables at the 10^th^, 25^th^, 50^th^, 75^th^, and 90^th^ percentiles. Predictor variables centered.

In order to explore the interaction, we used the PROCESS program [86] where moderation can be examined while including the 3 demographic factors (education, income, and sex) and the other stigma variables as controls. Figure 1 shows that as hypothesized, when the magnitude of the identity to the self is low – in this case when centrality is low–the level of internalization does not predict distress. However, as the level of centrality rises, the positive relationship of internalization to distress also rises. Another way to describe this result is that the relationship between internalization and distress does not become significant until centrality reaches the 75^th^ percentile (t = 2.30, p = .02). Thus, internalization of negative stereotypes about one's CSI is particularly distressing to the extent that the identity is also highly central to one's self-definition. We did not find any other significant two-way interactions, including between outness and anticipated stigma as we hypothesized.

### Examining the 5 CSI Groups

Thus far we have focused on examining a prediction model for the full sample of participants with CSIs. We now turn to examining the 5 specific identities. First, we will examine whether there are between group differences in the experience of the identities. We predicted that people with the more culturally stigmatized identities – mental illness and substance abuse – would anticipate and internalize more stigma than those with less culturally stigmatized identities – domestic violence, sexual assault, and childhood abuse. We made no a priori predictions for centrality, salience, or outness. Statistical significance of Bonferroni corrected post hoc tests are reported in the legend under each figure of means.

#### Anticipated stigma

**Figure 2:**
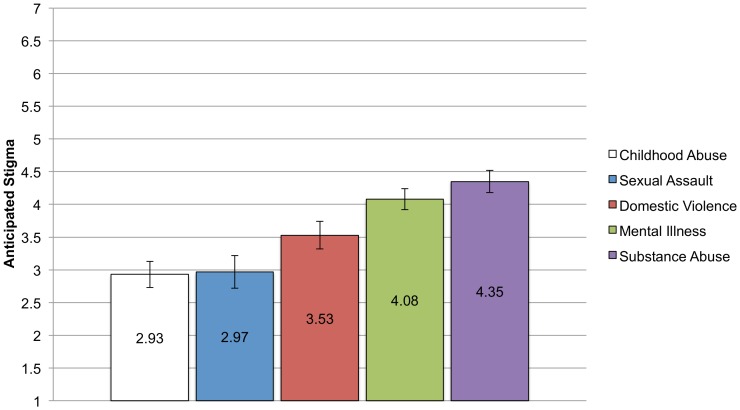
Means and Standard Errors for Anticipated Stigma for each CSI group. Analyses controlling for income, education, and sex found significant differences in mean levels of anticipated stigma reported across the groups. Bonferroni corrected post-hoc comparisons show that the substance abuse groups reported greater levels of anticipated stigma than the child abuse (p<.001), sexual assault (p<.001), and domestic violence groups (p = .03) but not more than the mental illness group. Similarly, the mental illness group reported more anticipated stigma than the child abuse (p<.001) and sexual assault groups (p = .003) but not the domestic violence group.

The means and standard errors for anticipated stigma for each CSI are shown in Figure 2. As hypothesized, participants in the mental illness and substance abuse groups reported the greatest amounts of anticipated stigma. A 1-way ANCOVA, covarying income, education, and sex was conducted with type of CSI (childhood abuse, sexual assault, domestic violence, mental illness, and substance abuse) as the between-subjects factor. The ANCOVA was significant, F (4, 383)  = 10.59, p<.001, η_p_^2^ = .10. Post-hoc comparisons revealed that the mean for the substance abuse group differed significantly from all other groups except the mental illness group. Similarly, the mental illness group differed from child abuse group and the sexual assault group but not the domestic violence group. The childhood abuse, sexual assault, and domestic violence groups did not differ from each other in level of anticipated stigma. The mental illness and substance abuse groups are above the midpoint of the scale, signifying they believe it to be likely that others will denigrate and socially distance from them if their CSI becomes known. The domestic violence mean is just under the midpoint of the scale, showing relative uncertainty about what might happen if they tell others.

#### Internalization

**Figure 3:**
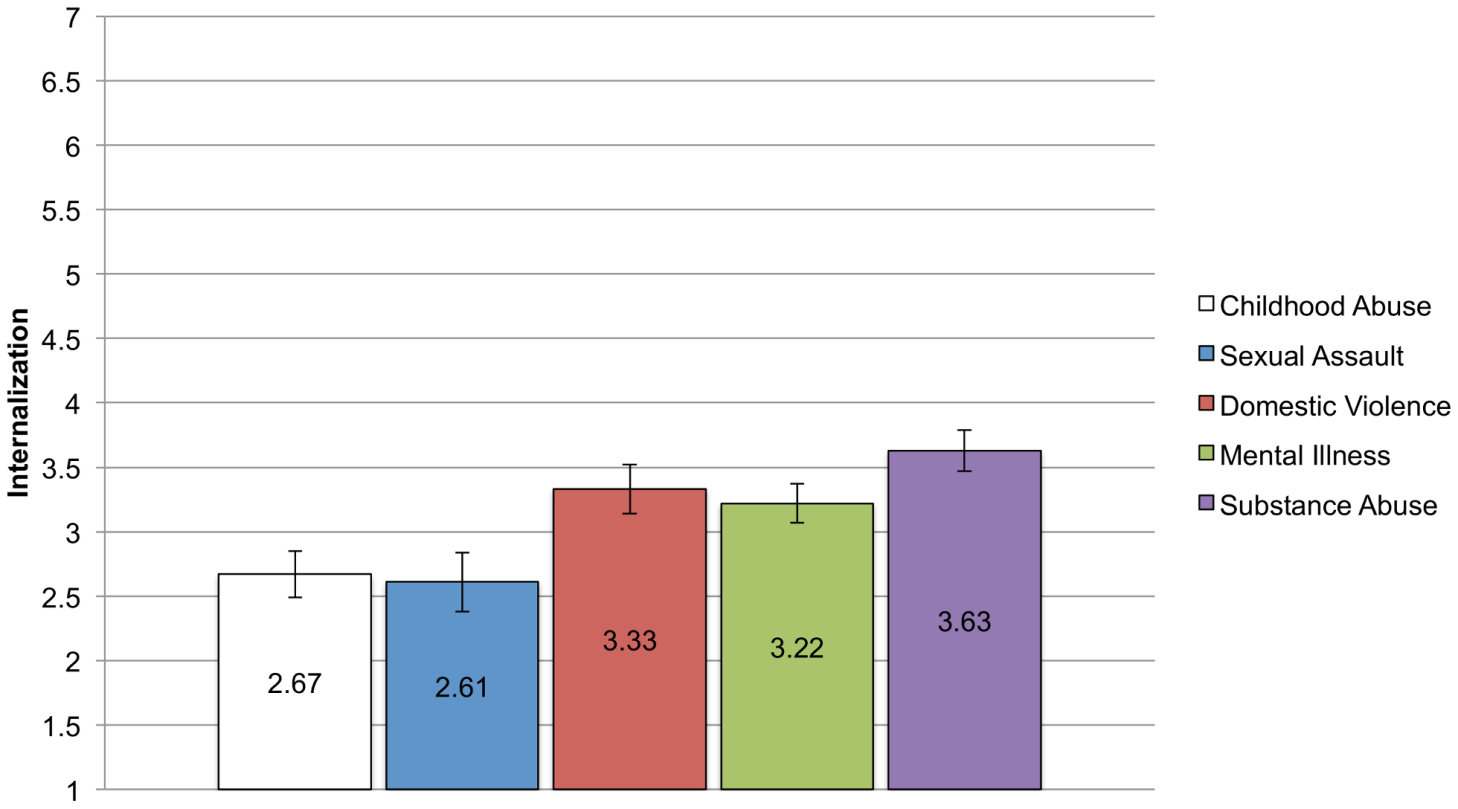
Means and Standard Errors for Internalization for each CSI group. Analyses controlling for income, education, and sex found significant differences in mean levels of internalization of stigma reported across the groups. Bonferroni corrected post-hoc comparisons show that the substance abuse groups reported greater levels of internalization than the child abuse (p = .001) and sexual assault groups (p = .003).

We hypothesized that the mental illness and substance abuse groups would report greater internalized stigma than the three other groups. Again, we conducted an ANCOVA, controlling for education, income, and sex, to examine between group differences. The ANCOVA was significant, F (4, 383)  = 5.71, p<.001, η_p_^2^ = .06. Post hoc tests gave partial support of our hypotheses. As can been seen in Figure 3, the substance abuse group reported the highest levels of internalization and they differed significantly from the two groups reporting the lowest levels of internalization: childhood abuse and sexual assault. The substance abuse group did not, however, differ from either the mental illness group or the domestic violence group. Indeed, there were no other differences between the groups. It is also notable that internalization is relatively low across the groups, signifying that people do not necessarily endorse negative stereotypes and beliefs about their identities.

#### Centrality and salience

**Figure 4:**
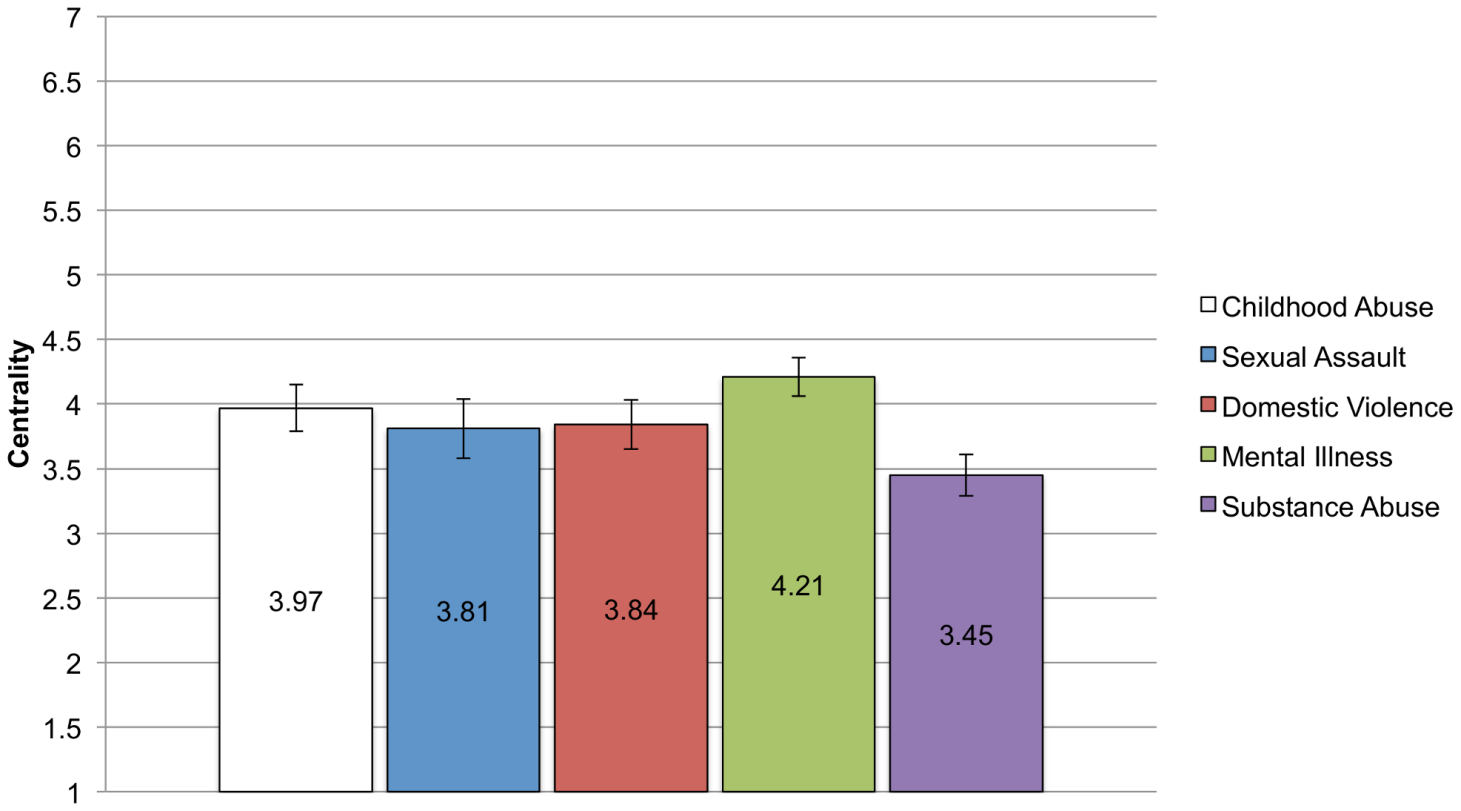
Means and standard errors for Centrality for each CSI Group. Analyses controlling for income, education, and sex found significant differences in mean levels of centrality reported across the groups. Bonferroni corrected post-hoc comparisons show that only the substance abuse and mental illness group report significantly different levels of centrality (p = .005). No other groups differ.

Examining centrality using ANCOVA with education, income and sex covaried, we find that the test of between group differences is significant F (4, 383)  = 3.27, p = .01, η_p_^2^ = .03, although the effect size is small. As can be seen in Figure 4, the group with the lowest level of centrality for the identity is the substance abuse group, which differed significantly from the mental illness group. There were no other differences between the groups. Thus despite being very different identities, with varying times of onset and continuation, the mean levels of centrality are similar and around the midpoint of the scale. It is of interest that the group that has the highest rates of internalization and anticipated stigma also reports that the identity is least self-definitional.

**Figure 5:**
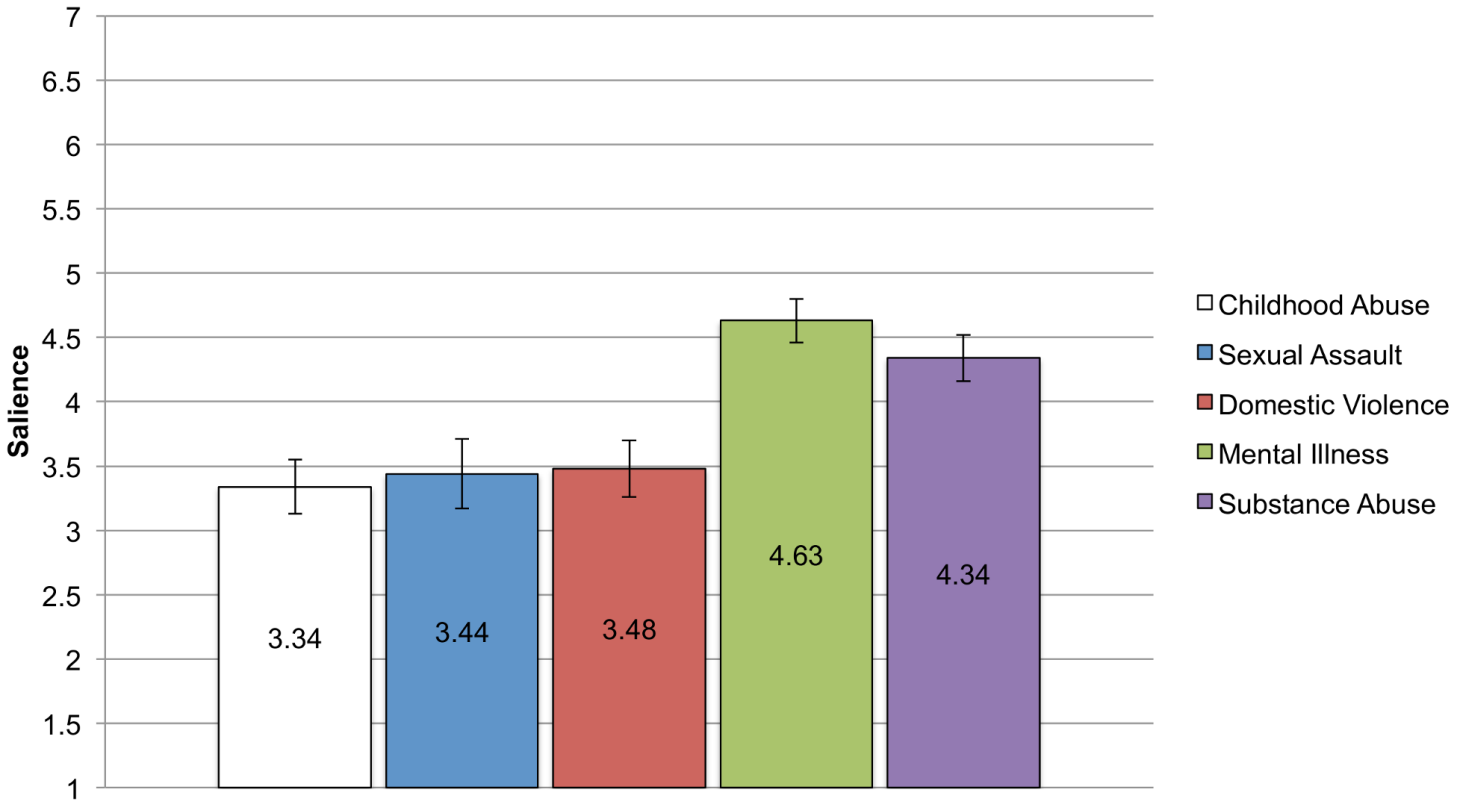
Means and standard errors for Salience for each CSI Group. Analyses controlling for income, education, and sex found significant differences in mean levels of salience reported across the groups. Bonferroni corrected post-hoc comparisons show that the mental illness group reports significantly higher identity salience than the childhood abuse (p<.001), sexual assault (p = .002), and domestic violence groups (p = .001), but no difference from the substance abuse group. Likewise, the substance abuse group differs from the childhood abuse (p = .003) and domestic violence groups (p = .03) although it is only marginally different from the sexual assault group (p = .07).

Figure 5 shows the means and standard errors for salience. The pattern here is different from centrality and more similar to anticipated stigma. Participants in the substance abuse and mental illness groups are reporting greater salience – more frequent thoughts about their identities – than participants in the childhood abuse, sexual assault, and domestic violence groups. As above, an ANCOVA covarying education, income, and sex shows a significant between groups effect, F (4, 383)  = 8.74, p<.001, η_p_^2^ = .08. Post-hoc comparisons show that the mental illness group reports significantly higher salience than the childhood abuse, sexual assault, and domestic violence groups, but no difference from the substance abuse group. Likewise, the substance abuse group differs from the childhood abuse and domestic violence groups although it is only marginally different from the sexual assault group. There are no significant differences in mean salience level between the childhood abuse, sexual assault, and domestic violence groups.

#### Outness

**Figure 6:**
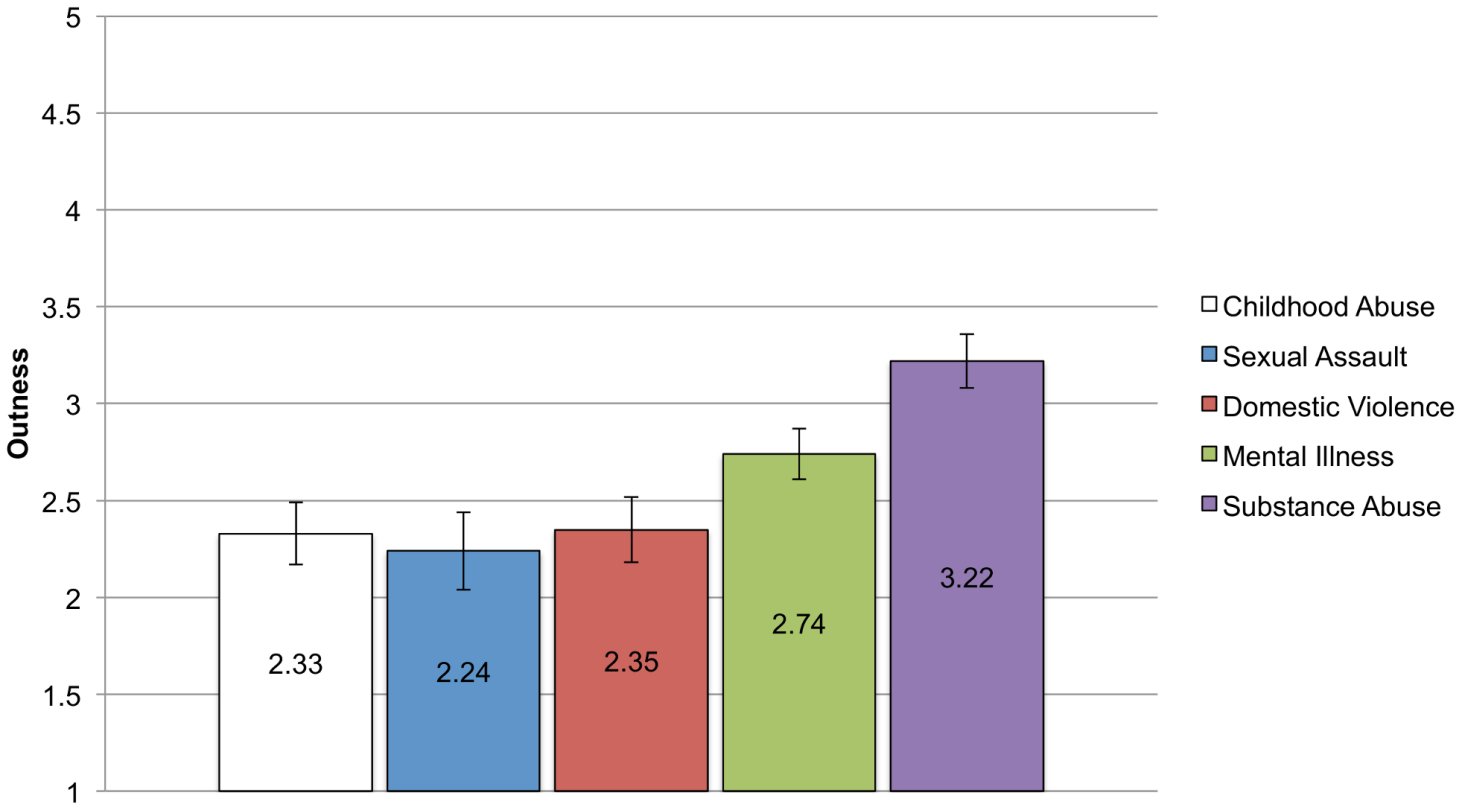
Means and standard errors for Outness for each CSI Group. Analyses controlling for income, education, and sex found significant differences in mean levels of outness reported across the groups. Bonferroni corrected post-hoc comparisons show that the substance abuse group reported being significantly more out about their identity than the childhood abuse (p<.001), sexual assault (p = .001), and domestic violence (p = .001) groups but not different from the mental illness group (p = .12). There were no other significant between group differences.

Figure 6 shows the means for outness by group. Surprisingly, the group that reported the most anticipated and internalized stigma also reported being the most out—the substance abuse group. An ANCOVA with the same covariates as above was significant, F (4, 383)  = 6.68, p<.001, η_p_^2^ = .07. Post hoc comparisons show that the substance abuse group was significantly more out than all other groups except the mental illness group. There were no other significant between group comparisons.

In summary, there were group differences in the mean level of the stigma variables, reflecting the different experiences and beliefs that people have surrounding their stigmatized identities. The effect size of group type on stigma variable was small, with group type accounting for between 3 and 8 percent of the variance in the stigma variables. As predicted, the substance abuse and mental illness groups had higher levels of anticipated stigma and internalization, but contrary to predictions, the substance abuse group reported being the most out but with the lowest level of identity centrality. Moreover, the domestic violence group had relatively high levels of internalization. The childhood abuse and sexual assault groups report the lowest levels of anticipated stigma, internalization, salience, and outness, but levels of identity centrality were on par with the other groups.

### Examining Moderation of the Prediction Model by Identity Type

We hypothesized that although the mean levels of the stigma variables might differ by identity type, the predictive model of distress would be similar across the identities. In order to examine this hypothesis, a nested, multi-group path model was tested to examine potential moderating effects of CSI group (utilizing AMOS 17). In this approach, the multivariate regression model is estimated separately for the five groups and the magnitude of the regression coefficients can be compared using a critical ratios z test [87]. Five models were tested against a default model. In the default model, all of the parameters of interest (i.e., regression paths between each of the independent variables and distress) were freely estimated for the five groups. This is identical to the regression model described above in Table 3, Step 4. Then, the default model was compared to models in which the path from one of the independent variables (e.g., anticipated stigma → distress) was constrained to be equal across the five stigmatized groups; this is repeated for each predictor variable. In this approach, the difference in chi square values between the constrained and unconstrained models indicates if the particular path is equivalent or different across the five groups. In cases when the chi-square differential (CMIN) was significant—signifying a moderation of the path by CSI group–follow-up pairwise contrasts were made using critical ratio z tests to see which specific groups differed on that path.

As would be expected given the linear regression above, the default model has excellent model fit in predicting distress, with a non-significant chi-square (*df* = 10)  = 14.32, *p* = .16; the comparative fit index (CFI)  = .991, and RMSEA = .033. Next, we examined the nested model comparisons. A significant chi-square differential (CMIN) indicates that the path between predictor variables and distress should not be constrained to be equal, i.e., the paths differ significantly by CSI group. The CMIN statistic indicates the paths from anticipated stigma → distress [CMIN (df = 4) =  10.22, p = .04]; from centrality → distress [CMIN (df = 4)  = 12.65, p = .01]; and from outness → distress [CMIN (df = 4)  = 9.62, p = .05] were moderated by CSI group; whereas the paths from salience → distress [CMIN (df = 4)  = 1.73, p = .79]; and internalization → distress [CMIN (df = 4)  = 5.21, p = .27] were not moderated by CSI group. In Table 4 we list the standardized regression weights by CSI group. Examination of the regression weights makes it is clear that one group – the sexual assault group – is quite different from the others and is the cause of the significant moderation effects. The regression weights for anticipated stigma, centrality, and outness are all in the opposite direction for the sexual assault group compared to the other 4 CSI groups, although only the centrality coefficient is significant (anticipated stigma is non-significant, and outness is marginal). Specifically, for the sexual assault group only, greater centrality of the identity is related to less distress, but greater outness is (marginally) related to more distress. Follow-up pairwise comparisons between each of the standardized regression weights support this finding. Using the critical ratio z-tests (at p<.05), for the anticipated stigma → distress path, the path for the sexual assault group is significantly different from each of the other groups, which do not differ from each other. For centrality → distress path, the sexual assault group differs from each of the other groups except substance abuse; none of the other groups differ from each other. And, for outness → distress, the sexual assault group differs from each of the other groups, which again do not significantly differ from each other.

**Table 4:** Predicting Psychological Distress Separately for each Concealable Stigmatized Identity Group.

	Substance Abuse (N = 104)	Mental Illness (N = 106)	Childhood Abuse (N = 78)	Domestic Violence (N = 67)	Sexual Assault (N = 49)
*Standardized Regression Weights*					
Income	−.12	−.22*	.07	−.23*	−.16
Education	−.13	−.02	−.14	−.07	−.04
Sex	.11	.06	.05	−.25*	.28*
Anticipated Stigma	.23*	.17	.30*	.27*	−.18
Centrality	−.03	.11	.16+	.19+	−.33*
Salience	.27*	.27*	.29*	.16	.39*
Internalization	−.07	.12	.17	.15	.26*
Outness	−.17+	−.20*	−.20*	−.12	.19+
**Total R^2^**	**.24**	**.36**	**.57**	**.53**	**.42**

Note: *p<.05, +p<.10.

To summarize, using a nested, multi-group path model to test for moderation by CSI group, we found that internalization and salience predict similarly across the 5 groups: greater salience is strongly related to greater distress; greater internalization is related to greater distress but the effect is relatively weak. Greater anticipated stigma is related to greater distress for the substance abuse, mental illness, childhood abuse, and domestic violence groups and these paths do not significantly differ across the 4 groups. However, for the sexual assault group, greater anticipated stigma is related to *less* distress (albeit not significantly). For outness, again, for the substance abuse, mental illness, childhood abuse, and domestic violence groups, more outness was related to less distress and the path weights did not significantly differ by group. For the sexual assault group, more outness was related to *more* distress. Finally, for centrality, level of centrality had weak to null predictive effects for substance abuse, mental illness, childhood abuse, and domestic violence groups but it was a strong negative predictor for the sexual assault group with greater centrality predicting lower levels of distress for this group only. Thus, for 4 of the CSI groups – substance abuse, mental illness, domestic violence, and childhood abuse – the prediction model works similarly, with no group moderation. However, the model works quite differently for the sexual assault group.

## Discussion

Using a diverse, low SES, urban sample of adults, we found that greater anticipated stigma from others, greater salience of the concealed stigmatized identity, greater internalization of negative beliefs about the identity, higher centrality of the stigmatized, and decreased outness were each correlated with greater distress. Salience, anticipated stigma, and outness each predicted a unique portion of variance in distress when entered simultaneously in regression. The full model accounted for just under 30% of the variance in distress, and the effects were not explained or weakened by the personality trait neuroticism/emotional stability. Moreover, we predicted that the magnitude of the identity might moderate the effects of negative beliefs and experiences around stigma – anticipated stigma and internalization – on distress. We did find a significant interaction between centrality of the identity and internalization such that when centrality was low – the identity was not considered self-definitional – internalization of negative beliefs about the identity were not related to distress. As the centrality of the identity to the self increased, greater internalization predicted greater distress. Thus, as predicted, to the extent that an identity has a greater magnitude within the self, negative beliefs related to the identity had a greater effect on outcomes. We also predicted, however, that anticipated stigma would moderate the positive effects of outness, similar to earlier work that found gay men who were the most rejection sensitive were hurt (rather than helped) by being more out about their identity [29]. However, we found no support for this prediction.

Because we targeted five specific CSIs – substance abuse, mental illness, domestic violence, sexual assault, and childhood abuse – we were also able to look for similarities and differences between these identities. Participants with a history of substance abuse reported the greatest levels of anticipated and internalized stigma. They also reported the greatest level outness and the lowest level of centrality. Although more work needs to be done, this seems like a particularly psychologically protective combination. That is, individuals with a history of substance abuse recognize that their identity may be devalued by others and may, in fact internalize that devaluation. And yet, the identity is not self-definitional and is less likely to be kept hidden from others. This pattern of response is consistent with the most common treatment modality for substance abuse—12 Step Fellowship. The 12-Step program emphasizes a certain degree of outness (e.g., attending meetings, public acknowledgement that one is an addict or alcoholic, seeking forgiveness for past wrongs, and working with a sponsor) but at the same time, there is a de-emphasis on attributing one's addiction to flawed character (e.g., addiction is a disease).

Participants with mental illness history show a slightly different pattern: they were relatively high in all of the stigma measures– anticipated stigma, internalization, centrality, salience, and outness. This pattern may make individuals with a history of mental illness particularly vulnerable to distress in that they are expecting stigma from others about an identity that they feel is self-definitional and for which they are more likely to endorse the negative stereotypes associated with it. People who experienced childhood abuse showed the lowest mean levels of anticipated and internalized stigma, but they did not differ from the other groups in terms of centrality of the identity. Finally, sexual assault and domestic violence showed similar mean levels of the stigma variables although the domestic violence group reported slightly (but not significantly) higher levels of anticipated and internalized stigma. Although we have focused on a general prediction model, we do not want to minimize the differences in the experiences of people with different identities.

We examined whether our prediction model was moderated by CSI group. We hypothesized that the prediction model would work similarly across the five groups. What we found was that for four of the groups – substance abuse, mental illness, domestic violence, and childhood abuse – the model did work the same, with anticipated stigma, internalization, and salience predicting more distress and outness predicting less distress (and centrality being non-significant). However, for the sexual assault group, analyses showed that internalization and salience continued to predict increased distress, but that greater anticipated stigma and greater centrality predicted *less* distress but greater outness predicted *more* distress. These results were unexpected and need to be replicated. The sexual assault group was our smallest group with only 49 participants and thus, we are cautious in interpreting these findings. A case could be made that the greater centrality reflects an integration of the sexual assault identity that is positive for mental health, but this does not easily concur with the idea that being more out to others would be psychologically risky. Certainly more work needs to be done to follow up and replicate these unexpected findings.

### Implications for Reducing Psychological Distress

Testing a model of concealable stigmatized identities has several important clinical implications. First and foremost is the opportunity to provide an integrated model of psychological vulnerability that takes into account individual, social, clinical, and socio-cultural factors. Based on lifetime prevalence rates, it is likely that most people will either experience a stigmatized identity or care for or about someone who does. Thus, it is critical to understand how stigmatization affects psychological well-being on both an individual and societal level. On a societal level, stigmatization may increase discriminatory behaviors, victimization, isolation, and alienation and continue to facilitate myths and stereotypes regarding mental illness, abuse, and addiction. On an individual level, stigmatizing identities may result in unnecessary suffering as they tend to limit help seeking behaviors [88] and reduce positive treatment effects [89]. More than a decade ago, the Surgeon General (1999) identified stigma as the single greatest barrier to addressing mental health issues and as a primary factor in contributing to racial/ethnic disparities in care, and yet most of the evidence-based interventions give scant attention to the role of stigma. For some individuals, concealing a stigmatized identity may be functional (i.e., situationally protective) even though the consequences of doing so may be maladaptive (i.e., increased symptomatology, limited support). Such issues may be particularly complicated for individuals managing multiple concealed stigmas (e.g., substance abuse and sexual assault), in conjunction with chronic or acute stressors (e.g., HIV positive status and poverty, depression and job loss), or both visible and concealed stigmatized identities (e.g., physical handicap and mental illness). Some recent interventions have begun to address ways to reduce self-stigma for people with serious mental illness [31], [90]–[91]. Specifically addressing stigma, particularly the degree to which is it self-definitional, internalized, salient, and frames expectations, may have a direct impact on treatment engagement and effectiveness.

The current work, however, has a number of limitations. We did not conduct formal diagnostic interviews and thus are not able to define the types of mental illness participants are experiencing. Also, we included only one personality variable as a control, but it is possible that there could be others that influence the perceptions of stigma and distress [92]. Because we are focused on creating a general model that can be used across multiple identities, we created or adapted measures that would work for multiple identities. Thus, we cannot test for content belief that is specific to a particular identity, such as knowledge of specific stereotypes about mental illness [93]–[94]. Also, because we asked participants to focus on one particular identity, we are not able to examine how they might feel about possessing multiple stigmatized identities or whether possessing multiple stigmatized identities affects the impact of the stigma variables. People with a stigmatized identity often have multiple identities and research examining how these identities intersect is very important although methodologically difficult (e.g., [95]–[96]). Finally, the sample sizes for our groups of sexual assault, domestic violence, and childhood abuse are relatively small. A larger, more nationally representative sample would strengthen conclusions to be drawn.

### Implications for Stigma Research

#### Replication and Generalization

Although university samples are convenient, it is important for stigma researchers to test their ideas on more diverse samples. College students have incredible resources in terms of access to healthcare and mental health professionals compared to most adults in the United States. Our sample was ethnically diverse and very low SES. Replicating research with different samples allows the researchers to see similarities and differences. In the current research we found partial replication of the research with college samples: In both samples, anticipated stigma and salience of the identity predict greater psychological distress. However, in the college sample, centrality of the identity also had a direct effect on distress. Although this direct effect did not replicate in our community sample, centrality did interact with internalization to predict distress. Another interesting comparison is that the mean anticipated stigma levels of the community sample are a full scale-point higher than the college samples. We believe this points to the reality of discrimination in the lives of members of the community sample. In summary, replication with multiple samples can increase the generalizability and reliability of stigma research, making researchers and clinicians more confident about the utility of the research.

#### Measuring Stigma

The current work is a step forward into specifying and testing five specific stigma constructs but more work needs to be done. We think it is particularly important to differentiate between internalized stigma, which is a belief that the negative stereotypes about the identity apply to the self, and anticipated stigma, which is a worry about being devalued once the identity is revealed. The primary stigma measure used in most research on concealed identities (e.g., mental illness stigma, HIV+ stigma, minority sexual orientation stigma) has been internalized stigma. Not surprisingly, internalized stigma correlates highly with low self-esteem and lower self-efficacy [23]. Anticipated stigma does not assume that people believe any negative stereotypes about their identity, yet in the current work anticipated stigma is a stronger predictor of psychological distress than internalized stigma.

Understanding the role of salience, separate from centrality, is an important future direction. In the current work, salience of the identity was the strongest predictor of distress. Future research could focus on whether heightened salience is due to frequent life disruptions due to such things as identity-related symptoms, required medication use, or treatment utilization; or whether salience is capturing the cognitive burden of holding an identity secret [38]. Continued refinement of the measurement of stigma constructs will help to determine which aspects of stigma are the most important as points of intervention.

## Conclusion

Understanding how possessing a concealable stigmatized identity impacts psychological distress is crucially important to the development of methods to alleviate distress and increase resilience. The current work highlights how identity beliefs and experiences at the individual level relate to distress. The individual level, however, must be combined with research examining ways to reduce discrimination at the institutional and interpersonal levels. Only then will the burden of stigma be lightened.

## Supporting Information

Text S1 **Experimenter Training.**
(DOCX)
