# Supplementary material for: Examining Effects of Anticipated Stigma, Centrality, Salience, Internalization, and Outness on Psychological Distress for People with Concealable Stigmatized Identities
Source: PLoS One. 2014 May 9;9(5):e96977. doi: 10.1371/journal.pone.0096977 (PMC4016201; doi:10.1371/journal.pone.0096977)
Supplement: Text S1 — Experimenter Training. (DOCX) [file pone.0096977.s001.docx]

**S1.**

**Experimenter Training.**

Because our study focused on identities that are both stigmatizing and concealable, considerable time was devoted to training and supervising research assistants to ensure that data were collected in a sensitive and consistent manner and that participant identities were not inadvertently exposed. Research assistants were trained using prepared scripts and role-playing prior to administering the study protocol. The role playing scenarios included ways to manage difficult participants, appropriate ways to decline individuals who were unable to participate due to impairment, lack of literacy, or previous participation, appropriate ways to recruit participants and interact with facility staff, and culturally responsive ways to assist participants who lacked familiarity with computers or research protocols (e.g., using Likert scales). Although the procedures were generally the same across all three data collection sites, some site-specific procedures were employed. For example, two of the sites provided mental and behavioral health services to individuals whose needs ranged from mild and acute to severe and chronic. As a result, only advanced graduate students, three of whom were advanced clinical students and two who were advanced social psychology students, collected data at these sites. All of the advanced clinical students had experience working in community or hospital-based mental health centers, had training in suicide assessment and crisis management as part of their program requirements, and were experienced at assessing clinical and psychological impairment. In all but two instances, an advanced clinical student was on-site for all data collection at these two sites. In addition, all of the research assistants at these sites participated in the facility’s standard orientation for new staff, which included building tours, security and emergency procedures, and crisis management. Research assistants met weekly (later biweekly) to check in and to discuss any issues related to the study or data collection. The study’s second author is a licensed clinical psychologist and served as the emergency contact and site supervisor for each of the data collection locations.

At each location one or more trained research assistants (always including a graduate assistant) approached people in public areas using a prepared script and wearing badges identifying their affiliation with the university conducting the research. Badges were used to ensure that participants did not mistake the research assistants as employees of the facility/location and to make clear that their participation would be voluntary. In the location that included mental and behavioral counseling, some participants were referred to the study by staff at the facilities. Staff members were trained to refer only people who were: (a) 18 years or older, (b) medically and psychologically stable, (c) not currently under the influence of any medications that might influence their ability to provide informed consent, and (d) participation was not considered contraindicated with their current treatment. At the community college site, some participants scheduled appointments to participate if they did not have time between classes to complete the study.

Because the survey was administered on a mini-laptop, participants needed to have some degree of computer literacy. The sample was very low income and ranged in age, and thus, many participants had limited experience using a computer and mouse (especially with a mini-laptop) or were unfamiliar with the format of some of the research questions (e.g., Likert scales). The research assistants received extensive training on how to provide instruction and assistance in culturally sensitive and unobtrusive ways. We received positive feedback from participants after the study noting that they enjoyed learning how to navigate around a computer and felt that the brief computer training was a benefit to them.
